# Improving Anticancer Activity of Doxorubicin by 4′-*epi*-Dehydroxyamination

**DOI:** 10.1021/acsmedchemlett.5c00681

**Published:** 2025-12-23

**Authors:** Anna A. Griadunova, Nicholas L. Petrone, Madeleine S. Maker, Brian Pallares, Trevor Leung, Allison N. Shim, Ömer H. Yilmaz, Jacob M. Goldberg, Jonathan Braverman, Fang Wang

**Affiliations:** ◪ Innovative Genomics Institute, 552695 University of California, Berkeley, Berkeley, California 94720, United States; ‡ Department of Chemistry, 4260 University of Rhode Island, Kingston, Rhode Island 02881, United States; § Department of Chemistry, 3719 Colgate University, Hamilton, New York 13346, United States; ∥ Department of Biology, The David H. Koch Institute for Integrative Cancer Research at MIT, 2167 Massachusetts Institute of Technology, Cambridge, Massachusetts 02139, United States; ⊥ Department of Pathology, Beth Israel Deaconess Medical Center, Massachusetts General Hospital and Harvard Medical School, Boston, Massachusetts 02215, United States

**Keywords:** anticancer, anthracyclines, doxorubicin, multidrug resistance, drug efflux, ABC transporters, P-glycoprotein

## Abstract

Efflux pump-mediated multidrug resistance is a common mechanism by which cancer cells reduce the efficacy of a broad range of small-molecule therapeutics. We discovered that substituting the 4′-hydroxy group of doxorubicin—a known efflux pump substrate—with an *epi*-amino group results in a new compound, doxorubamine, which exhibits substantially improved activity against drug-sensitive and -resistant cancer cells and organoids. Mechanistic studies reveal that doxorubamine is a poor substrate of P-glycoprotein, and it thus retains high potency against multidrug-resistant cancer. This synthetic modification provides a promising strategy for circumventing multidrug resistance beyond conventional approaches that rely on efflux pump inhibition.

## Graphical abstract

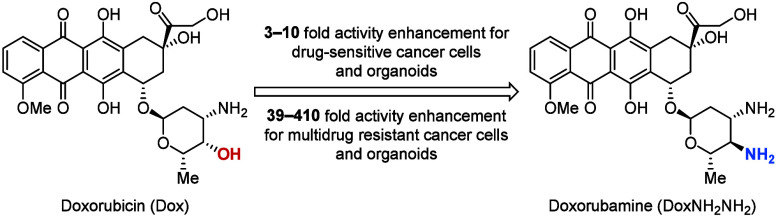

Drug resistance is a formidable barrier to effective cancer treatment. Among various mechanisms, multidrug resistance (MDR) is widely observed *in vitro* and is associated with the poor prognoses of different cancers.^1–10^ Extensive studies have shown that typical MDR is enabled by several ATP-binding cassette (ABC) transporters, including P-glycoprotein (P-gp), multidrug resistance-associated protein 1 (MRP1), and ATP-binding cassette superfamily G member 2 (ABCG2).^1,2,9,11,12^ The overexpression of these proteins, either as an intrinsic or acquired phenotype, leads to the undesired removal of a broad range of structurally unrelated small-molecule therapeutics from cancer cells, which reduces the efficacy of treatment. Conventional strategies for overcoming MDR primarily rely on inhibiting ABC transporters, thereby enhancing the retention of anticancer drugs by blocking efflux activity. This approach has led to the development of several generations of P-gp inhibitors with high *in vitro* activity. These inhibitors, however, confer limited clinical benefit or induce severe side effects.^1,9,13,14^ One problem arises from the need for inhibitors and anticancer drugs to act simultaneously in a particular tumor, thus requiring each agent to have similar pharmacokinetic profiles. Because nonmalignant tissues also express ABC transporters to clear endogenous and exogenous toxins, nonselective inhibition often causes adverse effects. Additional complications stem from the possible coexpression of multiple types of ABC transporters, which may require the use of several different inhibitors. These challenges underscore the need for new strategies to overcome MDR.

While studying transition metal-based agents for treating multidrug-resistant cancers,^15^ we synthesized doxorubamine (DoxNH_2_NH_2_), the 4′-*epi*-dehydroxyaminated derivative of doxorubicin (Dox), which is a classic efflux pump substrate (Figure 1).^16,17^ We serendipitously discovered that doxorubamine efficiently killed drug-sensitive human ovarian cancer A2780 cells and the corresponding multidrug-resistant A2780ADR cell line.^18^ Surprisingly, compared to various strategies to enhance the activity of anthracycline drugs,^19–27^ this relatively minor structural modification not only introduces a 10-fold increase in potency against A2780 cells but also enables doxorubamine to retain activity in efflux-high A2780ADR cells, which exhibit 410-fold resistance toward doxorubicin (Table 1, Entries 1 and 2, and Figure S5).

**Figure 1:**
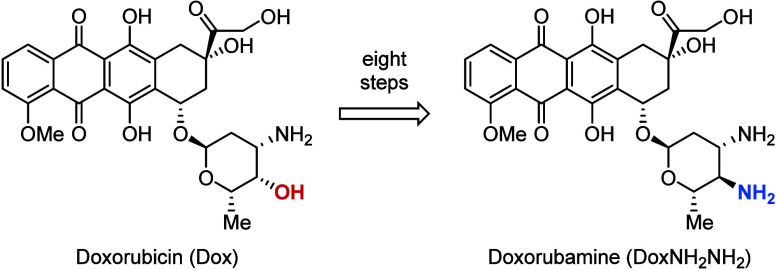
Molecular structures of doxorubicin (Dox) and doxorubamine (DoxNH_2_NH_2_).

**Table 1:** Comparison of the Cytotoxicity of Dox and DoxNH_2_NH_2_ against Various Cell Lines^a^

entry	treatment	IC_50_ ± (nM) for A2780	IC_50_ ± (nM) for A2780ADR	Fold resistance (IC_50,A2780ADR_ ÷ IC_50,A2780_)
1	Dox	8.4 ± 2.2	577 ± 349	69
2	DoxNH_2_NH_2_	0.83 ± 0.38	1.4 ± 0.6	1.7
3	Dox + verapamil	7.6 ± 2.1	25 ± 8	3.4
4	DoxNH_2_NH_2_ + verapamil	0.93 ± 0.14	0.46 ± 0.27	0.49
	Potency increase verapamil (−) (IC_50,Dox_÷IC_50,DoxNH_2_NH_2__)	10	410	
	treatment	IC_50_ ± (nM) for MES-SA	IC_50_ ± (nM) for MES-SA/Dx5	Fold resistance (IC_50,MES‑SA/Dx5_ ÷ IC_50,MES‑SA_)
5	Dox	12 ± 1	185 ± 61	15
6	DoxNH_2_NH_2_	2.0 ± 0.2	2.7 ± 0.1	1.3
7	Dox + verapamil	7.2 ± 0.4	42 ± 5	5.9
8	DoxNH_2_NH_2_ + verapamil	2.5 ± 0.3	2.3 ± 0.3	0.92
	Potency increase verapamil (−) (IC_50,Dox_÷IC_50,DoxNH_2_NH_2__)	6.0	68	
	treatment	IC_50_ ± (nM) for EL4	IC_50_ ± (nM) for EL4-DoxR	Fold resistance (IC_50,EL4‑DoxR_ ÷ IC_50,EL4_)
9	Dox	9.1 ± 0.9	1400 ± 50	155
10	DoxNH_2_NH_2_	2.8 ± 2.1	37 ± 3	13
11	Dox + tariquidar	3.3 ± 0.4	18 ± 2	5.3
12	DoxNH_2_NH_2_ + tariquidar	1.3 ± 0.1	2.6 ± 0.7	2.0
	Potency increase, tariquidar (−) (IC_50,Dox_ ÷ IC_50,DoxNH_2_NH_2__)	3.3	39	

a Experimental details can be found in the Supporting Information. Corresponding dose–response curves (*n* = 3 biological replicates) are shown in Figures S5 and S6.

Prompted by these observations, we evaluated the anticancer activity of doxorubamine with additional drug-sensitive and -resistant cell line pairs. As shown in Table 1, compared to Dox, DoxNH_2_NH_2_ was six times more potent against drug-sensitive human uterine sarcoma MES-SA cells. More importantly, although multidrug-resistant MES-SA/Dx5 cells^28,29^ exhibited 15-fold resistance toward doxorubicin, DoxNH_2_NH_2_ completely abrogated drug resistance (Table 1, Entries 5 and 6, and Figure S5). Given the broad clinical use of anthracyclines in lymphoma treatment, we generated a drug-resistant lymphoma cell line, EL4-DoxR, by exposing the parental mouse T-cell lymphoma cell line, EL4, to increasing concentrations of doxorubicin, resulting in 115-fold resistance. In contrast, although DoxNH_2_NH_2_ displayed some cross-resistance toward EL4-DoxR, DoxNH_2_NH_2_ maintained high activity, with 3.3- and 39-fold higher potency than Dox against EL4 and EL4-DoxR lines, respectively (Table 1, Entries 9 and 10, and Figure S5). Overall, these results suggest that the 4′-*epi*-dehydroxyamination of doxorubicin enhances both the anticancer capacity and the ability to circumvent efflux-pump-mediated chemoresistance.

We investigated whether DoxNH_2_NH_2_ kills MDR cancer cells independently of efflux pump activity. We first treated drug-sensitive A2780 cells with a combination of anticancer agents and verapamil (10 μM), a widely used P-gp inhibitor.^18,30,31^ As expected, verapamil did not affect the cytotoxicity of either doxorubicin or doxorubamine (Table 1, entries 1 vs 3 and 2 vs 4, and Figure S5). In contrast, in drug-resistant A2780ADR cells, verapamil sensitized doxorubicin by 23-fold but showed little effect on the potency of doxorubamine, suggesting that doxorubamine is a poor P-gp substrate. Similar results were observed with the MES-SA and MES-SA/Dx5 cell line pairs – although efflux inhibition by verapamil substantially reversed resistance to doxorubicin, it exhibited negligible effects on the activity of doxorubamine. Subsequent tests on EL4 and EL4-DoxR cells revealed that verapamil alone reduced the viability of these cells, presumably because of off-target interactions, including inhibition of calcium transport (Figure S6).^30^ We tested a more specific P-gp inhibitor, tariquidar (100 nM), in cotreatment studies.^32,33^ Under these conditions, a strong sensitization of approximately 80-fold was observed in EL4-DoxR cells treated with doxorubicin. Still, the resistance toward doxorubamine was reversed by only 14-fold, revealing the inefficient removal of doxorubamine by P-gp (Table 1, Entries 11 and 12, and Figure S5). Collectively, these findings indicate that DoxNH_2_NH_2_ overcomes MDR primarily because it is intrinsically a poor P-gp substrate.

Next, we assessed the activity of doxorubamine with genetically engineered mouse colorectal cancer organoids. Compared to conventional cell lines, these three-dimensional *in vitro* models are more physiologically relevant as they mimic the structure and function of *in vivo* tumors.^34^ We specifically generated a model harboring mutations commonly found in colon cancer, including *APC* ^*–/–*^; *KRAS* ^*G12D*^; *p53* ^*–/–*^; *SMAD4* ^*–/–*^ (AKPS) organoids. These organoids were also engineered to express either tdTomato (tdT) or zsGreen (zsG) to facilitate fluorescence microscopy-based *in vitro* coculture studies. We derived the doxorubicin-resistant variants of these organoids, AKPS-tdT-DoxR, by repeatedly exposing them to increasing concentrations of doxorubicin. In coculture experiments, although AKPS-zsGreen organoids were effectively killed by doxorubicin, the AKPS-tdT-DoxR variant exhibited more than 1000-fold resistance to doxorubicin (Figure 2A, Table 2, Entry 1, and Figures S7–S11). In the presence of verapamil, the activity of doxorubicin against AKPS-tdT-DoxR was enhanced by 44-fold, suggesting that P-gp overexpression is the primary contributor to the observed drug resistance. In contrast, compared to doxorubicin, doxorubamine effectively killed both drug-sensitive and -resistant organoids, showing a 5-fold and more than 200-fold enhancement in potency, respectively (Figure 2B, Table 2, Entry 2, and Figures S7–S11). Moreover, DoxNH_2_NH_2_ circumvented MDR in resistant organoids through a mechanism largely independent of P-gp activity, as indicated by the insignificant effect of verapamil cotreatment on efficacy (Figure 2B, Table 2, Entry 4, and Figures S7–S11). These results recapitulate the observation with human cell line pairs: the high cellular retention of doxorubamine across multiple model systems appears to be a general feature of this compound.

**Figure 2:**
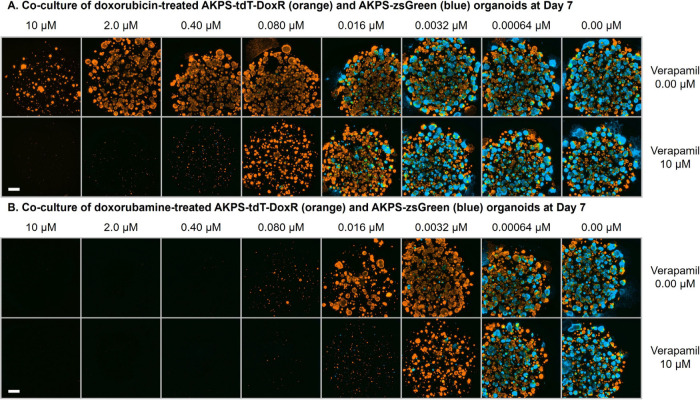
Representative fluorescence microscopic images showing the cocultured Dox-sensitive AKPS-zsG (blue) and Dox-resistant AKPS-tdT-DoxR (orange) cancer organoids treated with Dox or DoxNH_2_NH_2_ in the presence or absence of verapamil for 7 d. Scale bar = 500 μm. Experimental details can be found in the Supporting Information. Images of all three biological replicates are shown in Figures S8–S11.

**Table 2:** Comparison of the Cytotoxicity of Dox and DoxNH_2_NH_2_ against AKPS and AKPS-DoxR Cancer Organoids^a^

entry	treatment	IC_50_ ± SD (nM) for AKPS	IC_50_ ± (nM) for AKPS-DoxR	Fold resistance (IC_50,AKPS‑DoxR_ ÷ IC_50,AKPS_)
1	Dox	3.6 ± 2.6	4400 ± 500	1223
2	DoxNH_2_NH_2_	0.79 ± 0.20	20 ± 10	25
3	Dox + verapamil	12 ± 3	110 ± 20	9.0
4	DoxNH_2_NH_2_ + verapamil	0.95 ± 0.26	4.3 ± 1.9	4.5
	Potency increase verapamil (−) (IC_50,Dox_ ÷ IC_50,DoxNH_2_NH_2__)	4.6	220	

a Experimental details can be found in the Supporting Information. Corresponding dose–response curves (*n* = 3 biological replicates) are shown in Figure S7.

Finally, because both doxorubicin and doxorubamine are intrinsically fluorescent, we were able to compare the subcellular distribution of these two compounds in MES-SA cells using fluorescence microscopy (Figures S12–S18). As shown in Figure 3, both Dox and DoxNH_2_NH_2_ were distributed throughout the cytoplasm and nucleus. Fluorescence intensity quantification revealed that Dox-treated cells exhibited strong nuclear fluorescence. In contrast, DoxNH_2_NH_2_ showed more fluorescence in the cytoplasm. As anthracycline compounds commonly undergo fluorescence quenching upon DNA intercalation,^35–38^ these studies suggest that both Dox and DoxNH_2_NH_2_ primarily accumulate in the nucleus. Moreover, the logarithm of the partition coefficient (log *P*) values of Dox and DoxNH_2_NH_2_ in *n*-octanol–pH 7.4 phosphate buffer were −0.44 ± 0.02 and 0.12 ± 0.08, respectively, indicating that DoxNH_2_NH_2_ is only slightly more lipophilic or hydrophobic than Dox (Figure S19). Therefore, the substantially enhanced anticancer activity of doxorubamine may be attributable to factors other than alterations in subcellular distribution, cell permeability, lipophilicity, or hydrophobicity.

**Figure 3:**
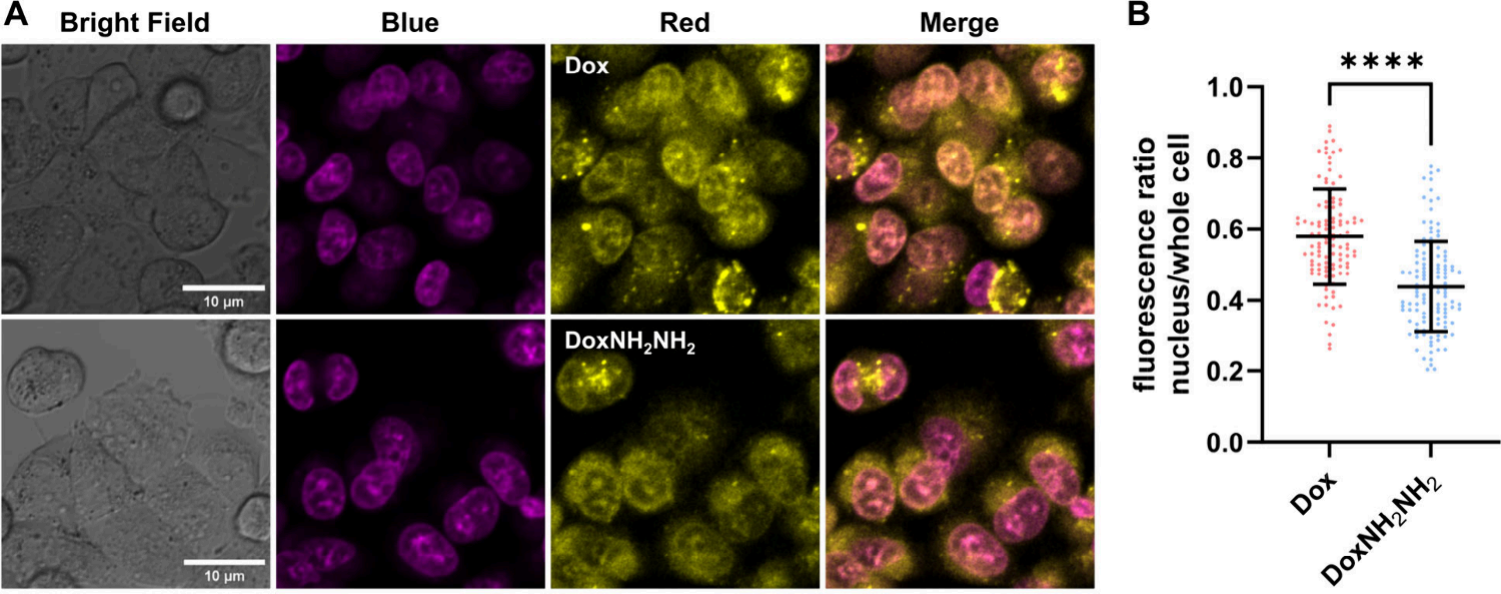
(A) Representative fluorescence images showing the subcellular distribution of doxorubicin (10 μM) or doxorubamine (10 μM) in MES-SA cells after 12 h treatment. The nuclei were stained with Hoechst 33342. Scale bar = 10 μm. Experimental details can be found in the Supporting Information. Images of two biological replicates are shown in Figures S12–S17. (B) Imaging quantification as measured by the ratio of fluorescence in the nucleus and in the whole cell. ****P < 0.0001 by unpaired two-tailed Welch’s *t* test. For doxorubicin, *n* = 106 cells. For doxorubamine, *n* = 128 cells. Error bars represent standard deviations.

In conclusion, converting the 4′-hydroxy group of doxorubicin into an *epi*-amino moiety substantially enhanced the anticancer activity of the resulting compound, doxorubamine, in both drug-sensitive and -resistant cancer models. Co-treatment experiments with P-gp inhibitors indicate that this structural modification renders doxorubamine a poor P-gp substrate, which allows doxorubamine to overcome efflux pump-mediated MDR. Fluorescence imaging studies reveal a similar subcellular distribution pattern for doxorubamine and the parent compound, doxorubicin, suggesting that the drastically varied bioactivity of doxorubamine may not be solely attributable to the difference in physical properties. Further investigations of the mode of action of doxorubamine and its mechanism of overcoming drug resistance are currently underway.

## Safety

*Caution!* Doxorubicin is probably carcinogenic to humans and must be handled with extreme care.

## Supplementary Material



## Notes

Detailed description of biological experiments, compound purity assessment by HPLC, dose–response curves for *in vitro* toxicity determination, images for organoid coculture experiments, images for compound subcellular distribution, and determination of partition coefficient (PDF)

## Abbreviations

Dox: doxorubicin
DoxNH_2_NH_2_: doxorubamine
IC_50_: half-maximal inhibitory concentration
MDR: multidrug resistance
ABC: ATP-binding cassette
P-gp: P-glycoprotein
MRP1: multidrug resistance-associated protein 1
ABCG2: ATP-binding cassette superfamily G member 2
APC: adenomatous polyposis coli
KRAS: Kirsten rat sarcoma virus
p53: cellular tumor antigen p53
SMAD4: Mothers against decapentaplegic homologue 4
AKPS: APC^–/–^
KRAS^G12D^: p53^–/–^
SMAD4^–/–^: organoids
tdT: tdTomato
zsG: zsGreen

## References

[1] Szakács G., Paterson J. K., Ludwig J. A., Booth-Genthe C., Gottesman M. M. (2006). Targeting Multidrug Resistance in Cancer. Nat. Rev. Drug Discovery.

[2] Shaffer B. C., Gillet J.-P., Patel C., Baer M. R., Bates S. E., Gottesman M. M. (2012). Drug resistance:
Still a daunting challenge to the
successful treatment of AML. Drug Resist. Updates.

[3] Holohan C., Van Schaeybroeck S., Longley D. B., Johnston P. G. (2013). Cancer Drug Resistance:
An Evolving Paradigm. Nat. Rev. Cancer.

[4] Szakács G., Hall M. D., Gottesman M. M., Boumendjel A., Kachadourian R., Day B. J., Baubichon-Cortay H., Di Pietro A. (2014). Targeting the Achilles Heel of Multidrug-Resistant
Cancer by Exploiting the Fitness Cost of Resistance. Chem. Rev..

[5] Gottesman M. M., Lavi O., Hall M. D., Gillet J.-P. (2016). Toward a Better
Understanding of the Complexity of Cancer Drug Resistance. Annu. Rev. Pharmacol. Toxicol..

[6] Wijdeven R. H., Pang B., Assaraf Y. G., Neefjes J. (2016). Old drugs, novel ways
out: Drug resistance toward cytotoxic chemotherapeutics. Drug Resist. Updates.

[7] Seluanov A., Gladyshev V. N., Vijg J., Gorbunova V. (2018). Mechanisms
of cancer resistance in long-lived mammals. Nat. Rev. Cancer.

[8] Ward R. A., Fawell S., Floc’h N., Flemington V., McKerrecher D., Smith P. D. (2021). Challenges and Opportunities
in Cancer
Drug Resistance. Chem. Rev..

[9] Robey R. W., Pluchino K. M., Hall M. D., Fojo A. T., Bates S. E., Gottesman M. M. (2018). Revisiting
the Role of ABC Transporters in Multidrug-Resistant
Cancer. Nat. Rev. Cancer.

[10] Tamaki A., Ierano C., Szakacs G., Robey R. W., Bates S. E. (2011). The controversial
role of ABC transporters in clinical oncology. Essays Biochem..

[11] Patch A.-M., Christie E. L., Etemadmoghadam D., Garsed D. W., George J., Fereday S., Nones K., Cowin P., Alsop K., Bailey P. J. (2015). Whole-genome characterization of chemoresistant
ovarian cancer. Nature.

[12] Robey R. W., Lusvarghi S., Chau C. H., Basseville A., Figg W. D., Ambudkar S. V., Bates S. E. (2022). ABCG2, the Breast
Cancer Resistance Protein (BCRP). Drug Transporters.

[13] Binkhathlan Z., Lavasanifar A. (2013). P-glycoprotein
Inhibition as a Therapeutic Approach
for Overcoming Multidrug Resistance in Cancer: Current Status and
Future Perspectives. Curr. Cancer Drug Targets.

[14] Palmeira A., Sousa E., H. Vasconcelos M., M. Pinto M. (2012). Three Decades of P-gp
Inhibitors: Skimming Through Several Generations and Scaffolds. Curr. Med. Chem..

[15] Wang F., Braverman J., Eng G., Leylek Ö., Petrone N. L., Honeycutt D. S., Imada S., Pallares B., Zhang D., Mrosla J. M. (2025). Leveraging platinum-protein
interactions to
overcome chemoresistance. Nat. Commun..

[16] Shen F., Chu S., Bence A. K., Bailey B., Xue X., Erickson P. A., Montrose M. H., Beck W. T., Erickson L. C. (2008). Quantitation of
Doxorubicin Uptake, Efflux, and Modulation of Multidrug Resistance
(MDR) in MDR Human Cancer Cells. J. Pharmacol.
Exp. Ther..

[17] Biedler J. L., Riehm H. (1970). Cellular Resistance to Actinomycin D in Chinese Hamster Cells in
Vitro: Cross-Resistance, Radioautographic, and Cytogenetic Studies1. Cancer Res..

[18] Rogan A., Hamilton T., Young R., Klecker R., Ozols R. (1984). Reversal of
adriamycin resistance by verapamil in human ovarian cancer. Science.

[19] Wander D. P. A., van der Zanden S. Y., Vriends M. B. L., van
Veen B. C., Vlaming J. G. C., Bruyning T., Hansen T., van der Marel G. A., Overkleeft H. S., Neefjes J. J. C. (2021). Synthetic (*N*, *N*-Dimethyl)­doxorubicin
Glycosyl Diastereomers to Dissect Modes of Action of Anthracycline
Anticancer Drugs. J. Org. Chem..

[20] van
Gelder M. A., Li Y., Wander D. P. A., Berlin I., Overkleeft H. S., van der Zanden S. Y., Neefjes J. J. C. (2024). Novel *N*, *N*-Dimethyl-idarubicin Analogues Are
Effective Cytotoxic Agents for ABCB1-Overexpressing, Doxorubicin-Resistant
Cells. J. Med. Chem..

[21] Priebe W., Perez-Soler R. (1993). Design and
tumor targeting of anthracyclines able to
overcome multidrug resistance: A double-advantage approach. Pharmacol. Ther..

[22] Perez-Soler R., Neamati N., Zou Y., Schneider E., Doyle L. A., Andreeff M., Priebe W., Ling Y. H. (1997). Annamycin
circumvents resistance mediated by the multidrug resistance-associated
protein (MRP) in breast MCF-7 and small-cell lung UMCC-1 cancer cell
lines selected for resistance to etoposide. Int. J. Cancer.

[23] Consoli U., Priebe W., Ling Y. H., Mahadevia R., Griffin M., Zhao S., Perez-Soler R., Andreeff M. (1996). The novel anthracycline annamycin is not affected by
P-glycoprotein-related multidrug resistance: comparison with idarubicin
and doxorubicin in HL-60 leukemia cell lines. Blood.

[24] Barbieri B., Giuliani F. C., Bordoni T., Casazza A. M., Geroni C., Bellini O., Suarato A., Gioia B., Penco S., Arcamone F. (1987). Chemical and Biological Characterization of 4’-Iodo-4’-deoxydoxorubicin. Cancer Res..

[25] Monneret C. (2001). Recent Developments
in the Field of Antitumour Anthracyclines. Eur.
J. Med. Chem..

[26] Mazel M., Clair P., Rousselle C., Vidal P., Scherrmann J.-M., Mathieu D., Temsamani J. (2001). Doxorubicin-peptide conjugates overcome
multidrug resistance. Anti-Cancer Drugs.

[27] Kolar C., Dehmel K., Moldenhauer H. (1990). Synthesis
of 4-*O*-methyl-β-rhodomycins using derivatives
of 4-amino-4-deoxy-
and 3,4-diamino-3,4-dideoxy sugars. Carbohydr.
Res..

[28] Wesolowska O., Paprocka M., Kozlak J., Motohashi N., Dus D., Michalak K. (2005). Human Sarcoma Cell Lines MES-SA and MES-SA/Dx5 as a
Model for Multidrug Resistance Modulators Screening. Anticancer Res..

[29] Harker W. G., Sikic B. I. (1985). Multidrug (Pleiotropic) Resistance in Doxorubicin-selected
Variants of the Human Sarcoma Cell Line MES-SA. Cancer Res..

[30] Tsuruo T., Iida H., Tsukagoshi S., Sakurai Y. (1981). Overcoming of Vincristine
Resistance in P388 Leukemia in Vivo and in Vitro through Enhanced
Cytotoxicity of Vincristine and Vinblastine by Verapamil. Cancer Res..

[31] Tsuruo T., Iida H., Tsukagoshi S., Sakurai Y. (1982). Increased Accumulation
of Vincristine and Adriamycin in Drug-resistant P388 Tumor Cells following
Incubation with Calcium Antagonists and Calmodulin Inhibitors. Cancer Res..

[32] Martin C., Berridge G., Mistry P., Higgins C., Charlton P., Callaghan R. (1999). The molecular interaction of the high affinity reversal
agent XR9576 with P-glycoprotein. Br. J. Pharmacol..

[33] Roe M., Folkes A., Ashworth P., Brumwell J., Chima L., Hunjan S., Pretswell I., Dangerfield W., Ryder H., Charlton P. (1999). Reversal of P-glycoprotein
mediated
multidrug resistance by novel anthranilamide derivatives. Bioorg. Med. Chem. Lett..

[34] Drost J., Clevers H. (2018). Organoids in cancer
research. Nat. Rev. Cancer.

[35] Mohan P., Rapoport N. (2010). Doxorubicin as a Molecular
Nanotheranostic Agent: Effect
of Doxorubicin Encapsulation in Micelles or Nanoemulsions on the Ultrasound-Mediated
Intracellular Delivery and Nuclear Trafficking. Mol. Pharmaceutics.

[36] Chaires J. B. (1983). Equilibrium
studies on the interaction of daunomycin with deoxypolynucleotides. Biochemistry.

[37] Airoldi M., Barone G., Gennaro G., Giuliani A. M., Giustini M. (2014). Interaction
of Doxorubicin with Polynucleotides. A Spectroscopic Study. Biochemistry.

[38] Pérez-Arnaiz C., Busto N., Leal J. M., García B. (2014). New Insights
into the Mechanism of the DNA/Doxorubicin Interaction. J. Phys. Chem. B.

