# Supplementary material for: Improving Anticancer Activity of Doxorubicin by 4′-epi-Dehydroxyamination
Source: ACS Med Chem Lett. 2025 Dec 23;17(1):48–53. doi: 10.1021/acsmedchemlett.5c00681 (PMC12794098; doi:10.1021/acsmedchemlett.5c00681)
Supplement: Supplementary file 1 [file ml5c00681_si_001.pdf]

Supporting Information

Improving anticancer activity of doxorubicin by 4'-*epi*-dehydroxyamination

Anna A. Griadunova,[1,†] Nicholas L. Petrone,[2,†] Madeleine S. Maker,[1,†] Brian Pallares,[3] Trevor Leung,[1] Allison N. Shim,[1] Ömer H. Yilmaz,[4,5] Jacob M. Goldberg,[3] Jonathan Braverman, [1,*] Fang Wang[2,*]

[1]Innovative Genomics Institute, University of California, Berkeley, Berkeley, CA 94720, USA
[2]Department of Chemistry, University of Rhode Island, 140 Flagg Rd, Kingston, RI 02881, USA
[3]Department of Chemistry, Colgate University, 13 Oak Drive, Hamilton, NY 13346, USA
[4]Department of Biology, The David H. Koch Institute for Integrative Cancer Research at MIT, Massachusetts Institute of Technology, Cambridge, MA 02139, USA
[5]Department of Pathology, Beth Israel Deaconess Medical Center, Massachusetts General Hospital and Harvard Medical School, Boston, MA 02215, USA
[†]These authors contributed equally.

*To whom correspondence should be addressed
E-mail: braverman@berkeley.edu; fangwang@uri.edu

Table of Contents

General 2
HPLC analysis showing the purity of the compounds used for this study 5
In vitro toxicity determination with conventional cancer cell lines 6
In vitro toxicity determination with co-cultured colon cancer organoids 8
Imaging studies of subcellular distribution 13
Determination of partition coefficient in n-octanol–pH 7.4 phosphate buffer mixture 21
Reference 22

General

Materials and analysis methods.

Pharmaceutical-grade doxorubicin hydrochloride (catalog # D1515) was purchased from Sigma-Aldrich. Doxorubicin stock solutions were prepared by dissolving doxorubicin hydrochloride in H_2O and stored at –20 °C. The stock solution of doxorubicin was used and discarded after each experiment. Doxorubamine diacetate was synthesized according to a reported procedure without modifications.[1] Doxorubamine diacetate stock solutions were prepared by dissolving doxorubamine acetate in H_2O and stored at –20 °C. Verapamil (Sigma-Aldrich catalog # V4629) was prepared as a 10 mM stock solution in 100% ethanol. Tariquidar (MedChemExpress, catalog # HY-10550) was prepared as a 1.0 mM stock solution in DMSO. The purity of all compounds used in biological studies was greater than 95%, as verified by HPLC.

Analytical HPLC was conducted using an Agilent 1200 Series system fitted with multi-wavelength detectors using a C18 reverse stationary phase (Zorbax-SB C18 column: 5 μm, 4.6 × 250 mm). The mobile phase was composed of two solvents – A: H_2O + 0.1% (v/v) CF_3CO_2H; B: CH_3CN + 0.1% (v/v) CF_3CO_2H, according to the following protocol: constant flow rate 1.0 $mL \cdot min^{-1}$; 0.0-5.0 min, 10% B; 5.0-30.0 min, linear gradient 10-100% B; 30.0-33.0 min, 100% B; 33.0-36.0 min, linear gradient 100-10% B; 36.0-40.0 min, 10% B.

UV-visible spectra were recorded on an Agilent Cary 60 UV-visible spectrophotometer. Cuvettes with 1.00 cm path lengths were used for all spectroscopic measurements. Milli-Q purified water, with a resistivity of at least 18 $M\Omega \cdot cm^{-1}$, was used to prepare all aqueous stock solutions.

Materials and methods for biological studies.

Cells and organoids were maintained at 37 °C and under a humidified 5% CO_2. Cell culture media were prepared with Advanced DMEM/F-12 (ThermoFisher catalog # 12634010), supplemented with fetal bovine serum (FBS, 5 vol %, HyClone™, Cytiva catalog # SH30910.03), 2 mM GlutaMAX™ (1 vol %, ThermoFisher catalog # 35050061), and 100 U/mL penicillin-streptomycin (1 vol %, 10,000 U/mL, ThermoFisher catalog # 15140122). Matrigel® (Corning catalog # 354230) was used as a 67 vol % mixture with cell culture media. Resazurin was purchased from ThermoFisher (catalog # R12204).

Cell lines and cytotoxicity determination with cell lines

The human ovarian cancer A2780 cell line was obtained from the Robert A. Swanson (1969) Biotechnology Center at the Massachusetts Institute of Technology. Cell lines, including A2780ADR (catalog # 93112520), MES-SA (catalog # 95051030), and MES-SA/Dx5 (catalog # 95051031), were purchased from Sigma-Aldrich. The Mouse T-cell lymphoma cell line EL4 (catalog # TIB-39) was purchased from ATCC. The doxorubicin-resistant EL4 cell line (EL4-DoxR) was generated by repeatedly treating the EL4 T-cell lymphoma cell line with increasing concentrations of doxorubicin until at least a 30-fold increase in IC_{50} was observed.

Organoid lines were generated in-house from colon crypts from mice on the C57BL/6 background, and the subsequent genetic modifications were validated by PCR. Cell lines from ATCC and Sigma-Aldrich were authenticated by the manufacturers using short tandem repeat (STR) profiling. These data can be found on the manufacturers' websites.

Cells were grown in media (10 mL) in 10 cm tissue culture plates. The cells were trypsinized and seeded into 96-well plates with 5×10^3 cells per well. Cells were treated with cytotoxic drug-containing media (200 µL) at the time of plating. For experiments with P-gp inhibition, verapamil or tariquidar was applied at the indicated concentration at the time of cell seeding, along with the cytotoxic agent. The cells were then incubated for six days following the drug treatment. Cell growth was measured by a resazurin assay. In brief, at the end of six-day drug treatment, resazurin was added to the cells at a final concentration of 50 µg/mL. The baseline fluorescence was determined immediately. The cells were then incubated at 37 °C for 2 h. The fluorescence of the resorufin product was measured at 560/590 nm and baseline corrected. The cell growth at each drug concentration was normalized to the untreated control.

Colorectal cancer organoids and cytotoxicity determination with cancer organoids

To assess the potency and efflux sensitivity of doxorubicin and doxorubamine in an *in vitro* solid tumor model, doxorubicin-resistant $APC^{-/-}$; $KRAS^{G12D}$; $p53^{-/-}$; $SMAD4^{-/-}$; $tdTomato^{+}$ (AKPS-tdT-DoxR) mouse colon cancer organoids[1] were co-cultured with non-resistant control $APC^{-/-}$; $KRAS^{G12D}$; $p53^{-/-}$; $SMAD4^{-/-}$; zsGreen (AKPS-zsG) organoids and treated with doxorubicin or doxorubamine with or without the efflux pump inhibitor verapamil (0, 0.4, 2.0, or 10 μM).

All experiments were performed in 48-well plates, with each well containing one Matrigel® droplet (9 μL, a mixture of 75 vol % Matrigel® and 25 vol% media with cells). After a 20 min-incubation at 37 °C for gelation, 450 μL of media (Advanced DMEM/F-12 with 5 vol % FBS, 1 vol % GlutaMAX, and 100 U/mL penicillin-streptomycin) containing anticancer agents with and without verapamil was added to each well. Co-cultures of AKPS-tdT-DoxR and AKPS-zsG organoids were grown in Matrigel® droplets and incubated at 37 °C for seven days. The organoids were imaged longitudinally on an ImageXpress Confocal HT.ai High-Content Imaging System. Images were acquired at 4× magnification using the FITC and Texas Red laser lines. One field of view was acquired per well, with 10 z-slices spanning 810 microns.

Two independent experiments were performed – one with 2,000 total cells per droplet from trypsinized AKPS-tdT-DoxR and AKPS-zsG organoids at a 1:1 ratio, and a second experiment with 4,000 total cells from trypsinized AKPS-tdT-DoxR and AKPS-zsG at a 3:1 ratio. For each experiment, a total of 192 organoid co-cultures were treated. Doxorubicin and doxorubamine were assessed at the following concentrations in triplicate: 10 μM, 2 μM, 400 nM, 80 nM, 16 nM, 3.2 nM, 0.64 nM, and 0.0 nM. These dose response experiments with doxorubicin and doxorubamine were performed at four different concentrations of verapamil (10 μM, 2.0 μM, 0.4 μM, and 0.0 μM).

To quantify the activity of anticancer agents, maximum intensity projection images were generated from the 10 z-planes captured for each well at each indicated time point. These image projections were then analyzed in FlowJo™ v10. To correct for dose-dependent drug fluorescence, median background subtraction was performed for each well and channel. Specifically, the median background fluorescent intensity per pixel of each channel and well was calculated. This value was subtracted from every pixel within that well.

To quantify organoid fluorescence, pixels were gated based on the histogram of each channel (FITC and Texas Red). The FITC-positive and Texas Red-positive populations were plotted on a Texas Red versus FITC scatter diagram to gate green, red, and double-positive pixels, corresponding to AKPS-zsG organoids, AKPS-tdT-DoxR organoids, and overlapping organoids, respectively.

For each color channel, the number of positive pixels was multiplied by the average intensity of these pixels to generate a single value representing the total fluorescence of the corresponding organoid genotype in each well at each time point.

HPLC analysis showing the purity of the compounds used for this study

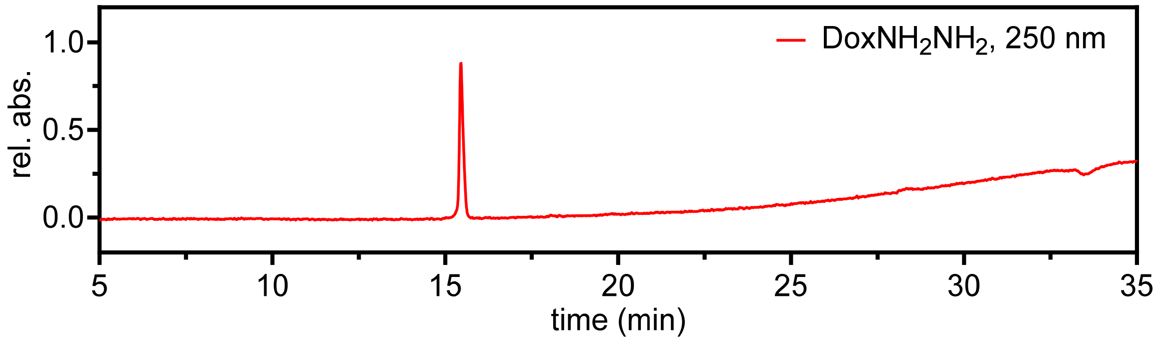

Figure S1. Analytical HPLC chromatogram of doxorubamine monitored at 250 nm. No detectable impurities were observed. t_R = 15.5 min.

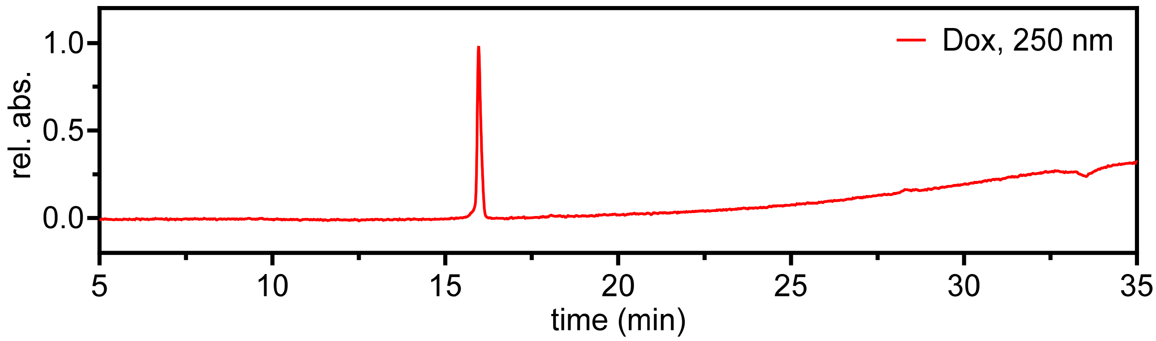

Figure S2. Analytical HPLC chromatogram of doxorubicin monitored at 250 nm. No detectable impurities were observed. t_R = 16.0 min.

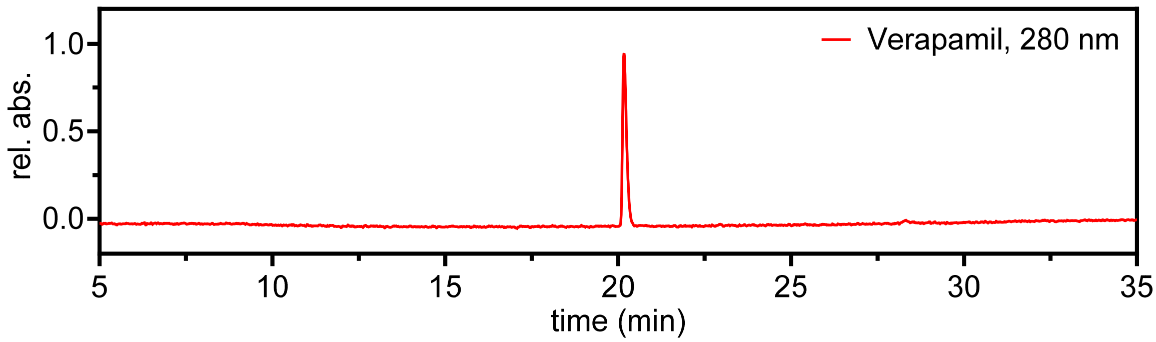

Figure S3. Analytical HPLC chromatogram of verapamil monitored at 280 nm. No detectable impurities were observed. t_R = 20.2 min.

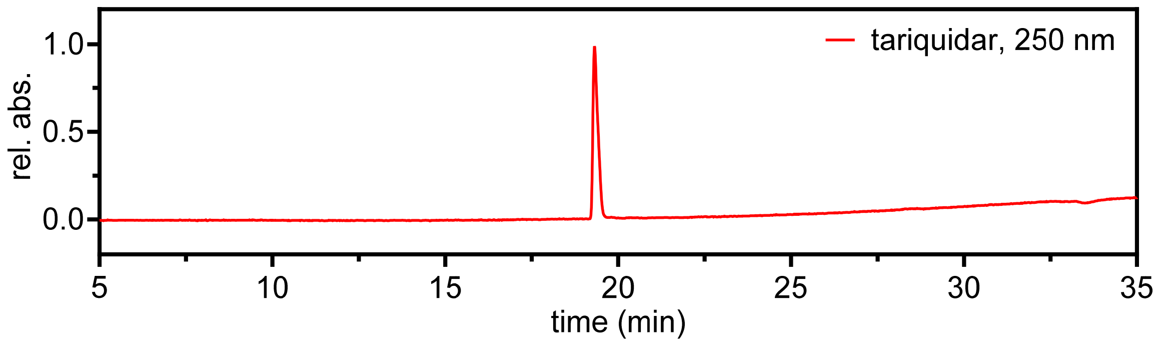

Figure S4. Analytical HPLC chromatogram of tariquidar monitored at 250 nm. No detectable impurities were observed. t_R = 19.3 min.

In vitro toxicity determination with conventional cancer cell lines

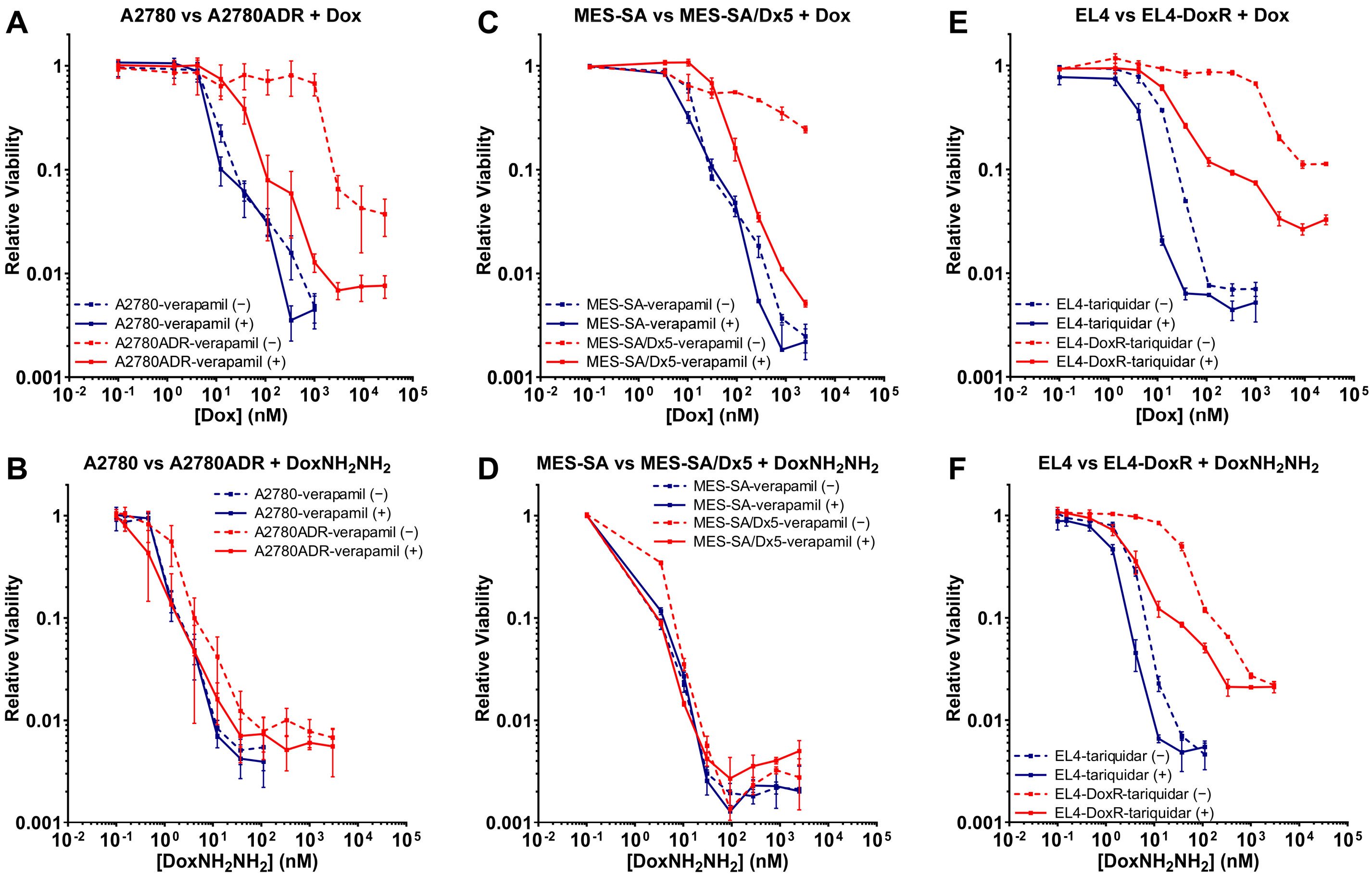

Figure S5. (**A**) Dose-response curves (n = 3 biological replicates) for Dox with A2780 human ovarian cancer cells (dashed blue line, without verapamil, and solid blue line, with 10 µM verapamil) and the doxorubicin-resistant variant A2780ADR (dashed red line without verapamil, and solid red line with 10 µM verapamil). (**B**) Dose-response curves (n = 3 biological replicates) for $DoxNH_2NH_2$ with A2780 human ovarian cancer cells (dashed blue line, without verapamil, and solid blue line, with 10 µM verapamil) and the doxorubicin-resistant variant A2780ADR (dashed red line without verapamil, and solid red line with 10 µM verapamil). (**C**) Dose-response curves (n = 3 biological replicates) for Dox with MES-SA human uterine sarcoma cells (dashed blue line, without verapamil and solid blue line, with 10 µM verapamil) and the doxorubicin-resistant variant MES-SA/Dx5 (dashed red line, without verapamil and solid red line, with 10 µM verapamil). (**D**) Dose-response curves (n = 3 biological replicates) for $DoxNH_2NH_2$ with MES-SA human uterine sarcoma cells (dashed blue line, without verapamil and solid blue line, with 10 µM verapamil) and the doxorubicin-resistant variant MES-SA/Dx5 (dashed red line, without verapamil and solid red line, with 10 µM verapamil). (**E**) Dose-response curves (n = 3 biological replicates) for Dox with mouse T-cell lymphoma EL4 cells (dashed blue line, without tariquidar, and solid blue line, with 100 nM tariquidar) and the doxorubicin-resistant variant EL4-DoxR (dashed red line, without tariquidar, and solid red line, with 100 nM tariquidar). (**F**) Dose-response curves (n = 3 biological replicates) for $DoxNH_2NH_2$ with mouse T-cell lymphoma EL4 cells (dashed blue line, without tariquidar, and solid blue line, with 100 nM tariquidar) and the doxorubicin-resistant variant EL4-DoxR (dashed red line, without tariquidar, and solid red line, with 100 nM tariquidar). Error bars represent standard deviation.

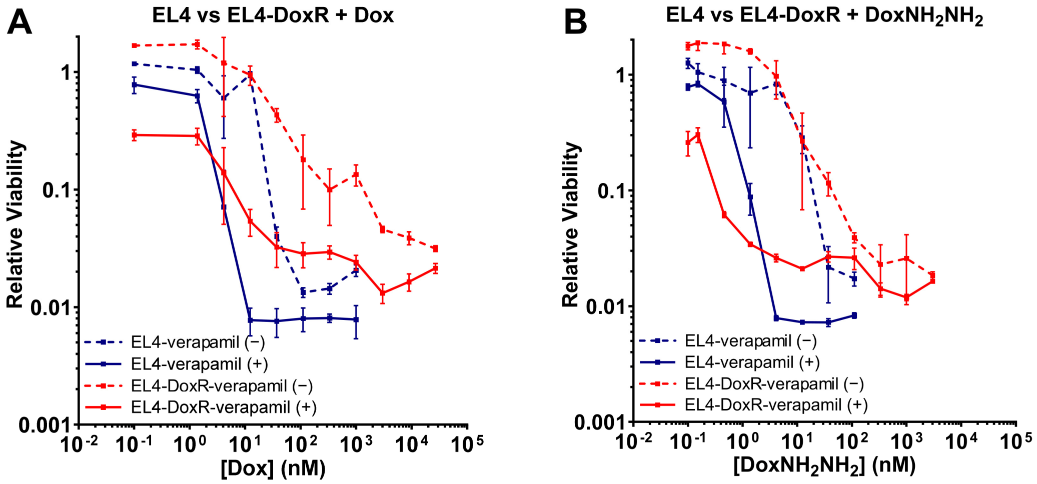

Figure S6. (**A**) Dose-response curves (*n* = 3 biological replicates) for Dox with mouse T-cell lymphoma EL4 cells (dashed blue line, without verapamil, and solid blue line, with 10 µM verapamil) and the doxorubicin-resistant variant EL4-DoxR (dashed red line, without verapamil, and solid red line, with 10 µM verapamil). (**B**) Dose-response curves (*n* = 3 biological replicates) for $DoxNH_2NH_2$ with mouse T-cell lymphoma EL4 cells (dashed blue line, without verapamil, and solid blue line, with 10 µM verapamil) and the doxorubicin-resistant variant EL4-DoxR (dashed red line, without verapamil, and solid red line, with 10 µM verapamil). Error bars represent standard deviation.

In vitro toxicity determination with co-cultured colon cancer organoids

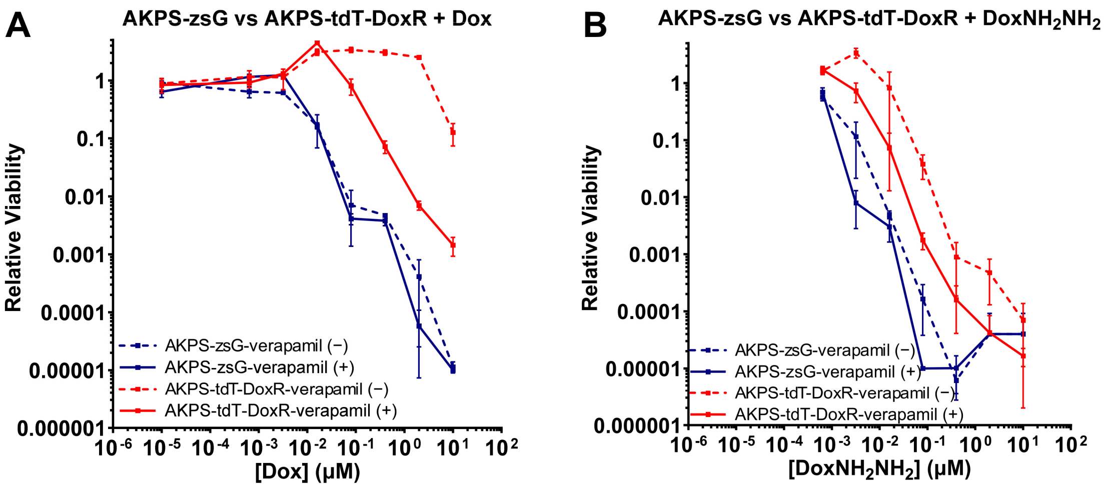

Figure S7. Quantifications of images of AKPS-zsG and AKPS-tdT-DoxR 1:1 co-cultures in the form of dose-response curves. (**A**) Dose-response curves (n = 3 biological replicates) for Dox with AKPS-zsG colon cancer organoids (dashed blue line, without verapamil, and solid blue line, with 10 μM verapamil) and the doxorubicin-resistant variant AKPS-tdT-DoxR (dashed red line, without verapamil, and solid red line, with 10 μM verapamil). (**B**) Dose-response curves (n = 3 biological replicates) for $DoxNH_2NH_2$ with AKPS-zsG colon cancer organoids (dashed blue line, without verapamil, and solid blue line, with 10 μM verapamil) and the doxorubicin-resistant variant AKPS-tdT-DoxR (dashed red line, without verapamil, and solid red line, with 10 μM verapamil). Error bars represent standard deviation.

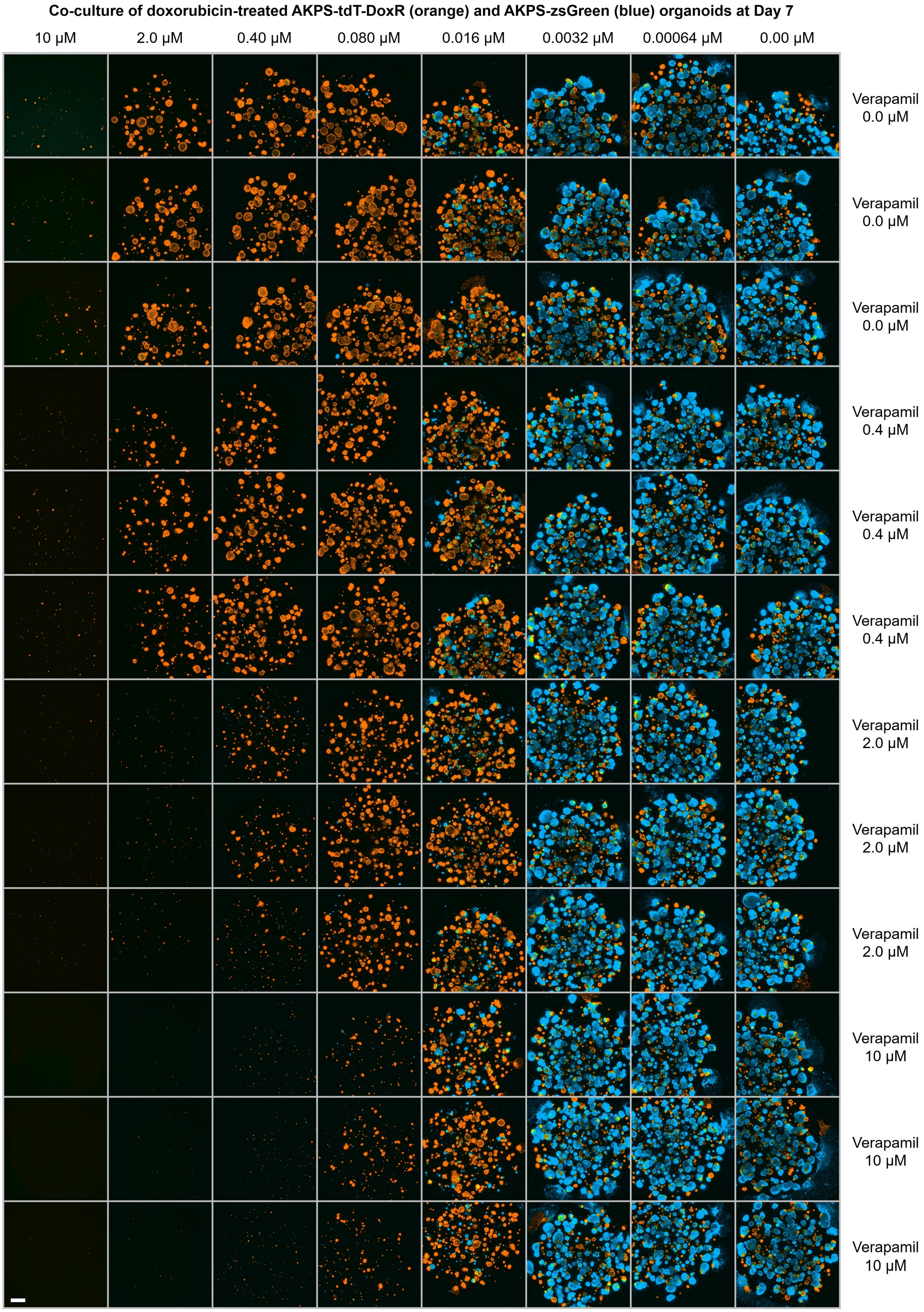

Figure S8. Images from fluorescence microscopy studies showing the anticancer activity of doxorubicin against a co-culture of AKPS-zsG colon cancer organoids (blue) and the doxorubicin-resistant variant AKPS-tdT-DoxR (orange). The experiment was performed with 2,000 total cells per droplet from trypsinized AKPS-tdT-DoxR and AKPS-zsG organoids at a 1:1 ratio. Scale bar = 500 µm.

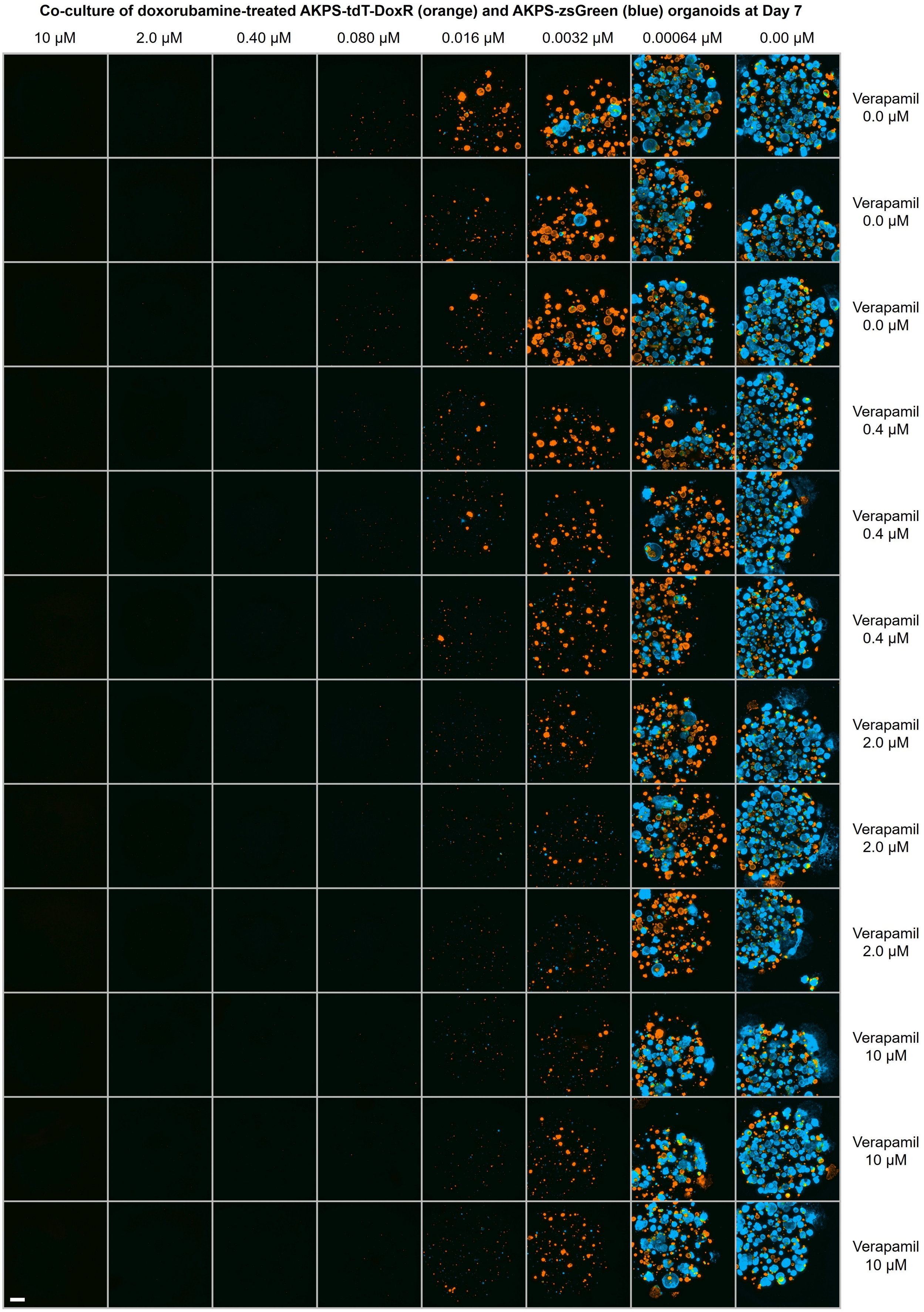

Figure S9. Images from fluorescence microscopy studies showing the anticancer activity of doxorubamine against a co-culture of AKPS-zsG colon cancer organoids (blue) and the doxorubicin-resistant variant AKPS-tdT-DoxR (orange). The experiment was performed with 2,000 total cells per droplet from trypsinized AKPS-tdT-DoxR and AKPS-zsG organoids at a 1:1 ratio. Scale bar = 500 µm.

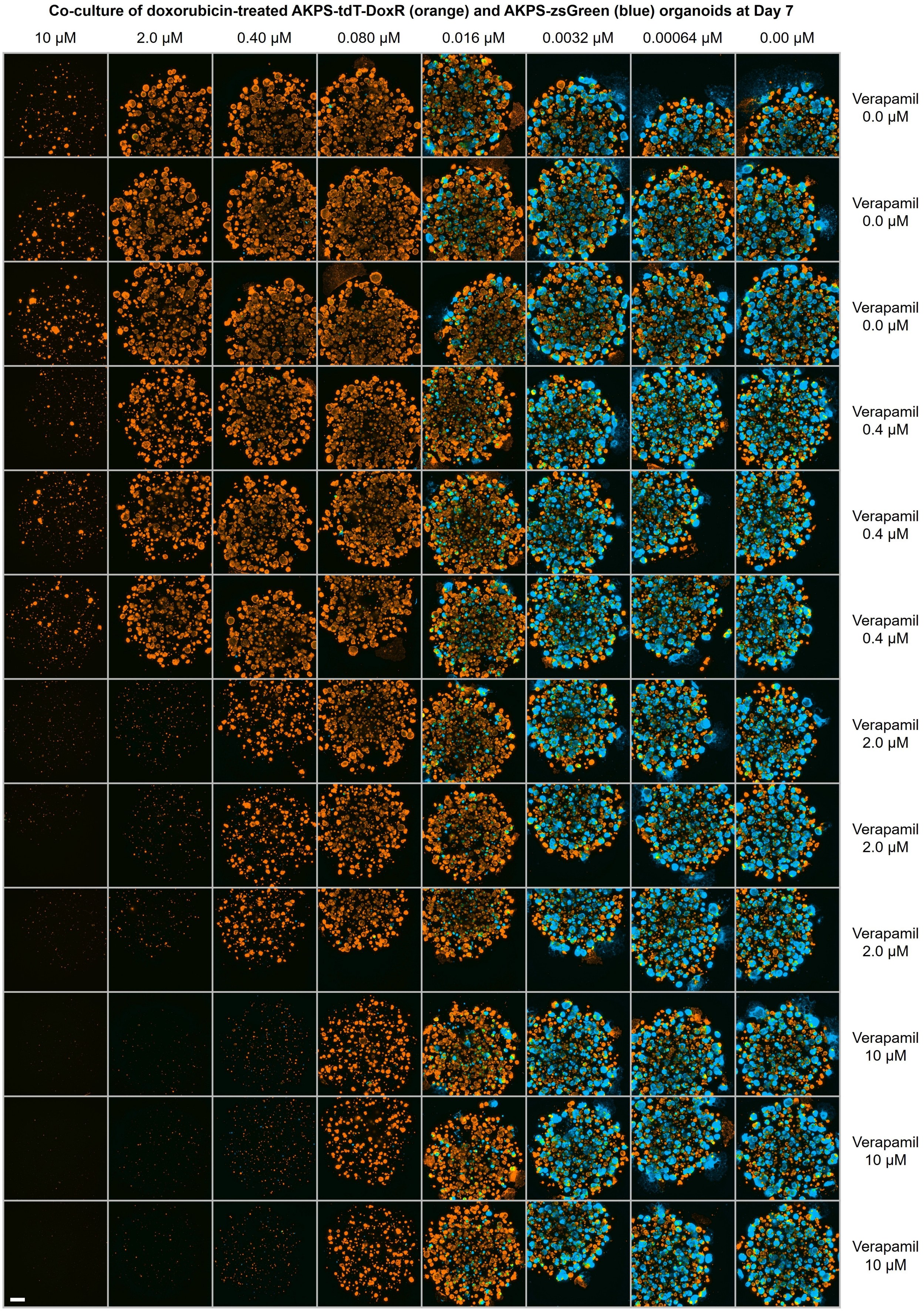

Figure S10. Images from fluorescence microscopy studies showing the anticancer activity of doxorubicin against a co-culture of AKPS-zsG colon cancer organoids (blue) and the doxorubicin-resistant variant AKPS-tdT-DoxR (orange). The experiment was performed with 4,000 total cells per droplet from trypsinized AKPS-tdT-DoxR and AKPS-zsG organoids at a 3:1 ratio. Scale bar = 500 μm.

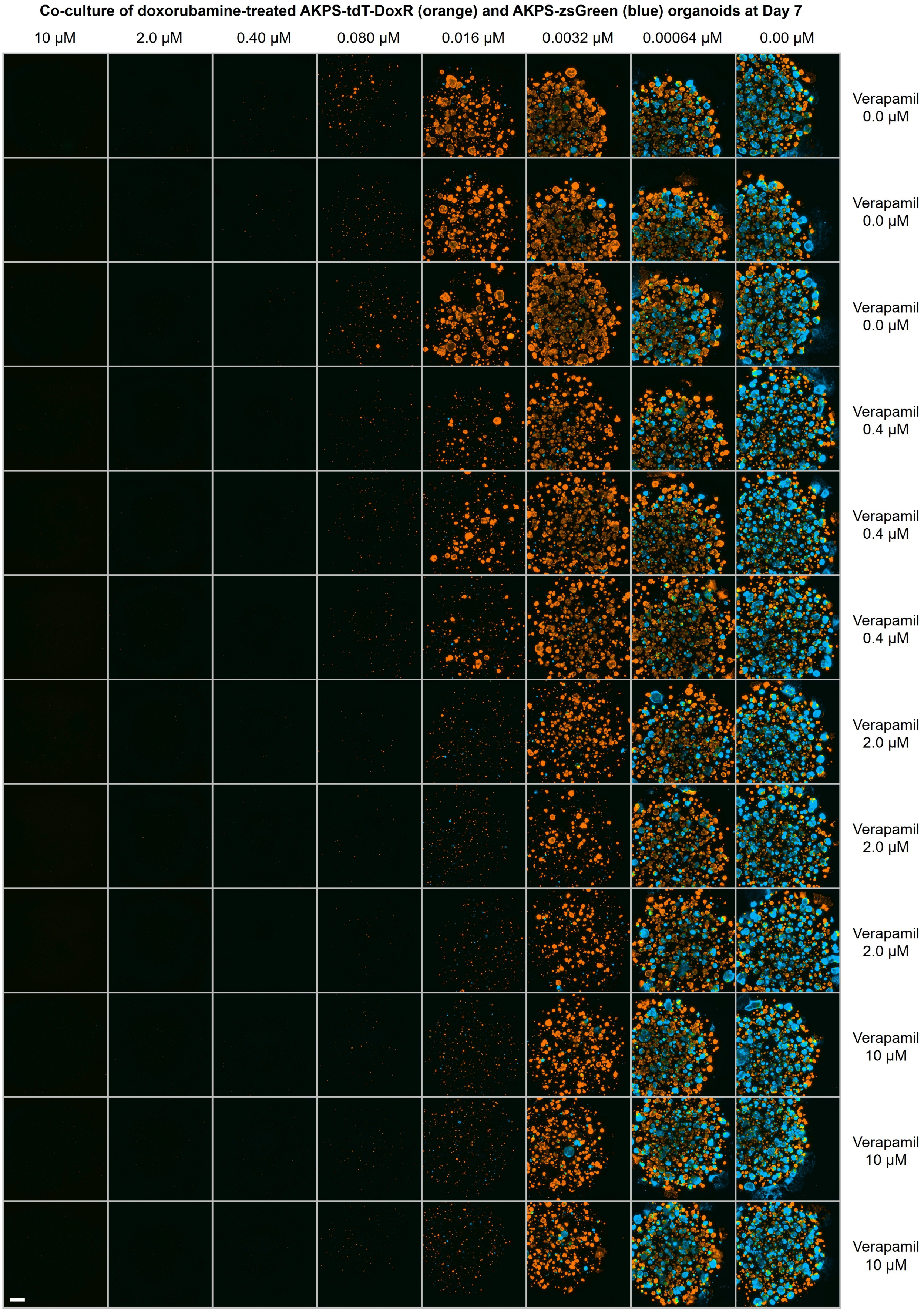

Figure S11. Images from fluorescence microscopy studies showing the anticancer activity of doxorubamine against a co-culture of AKPS-zsG colon cancer organoids (blue) and the doxorubicin-resistant variant AKPS-tdT-DoxR (orange). The experiment was performed with 4,000 total cells per droplet from trypsinized AKPS-tdT-DoxR and AKPS-zsG organoids at a 3:1 ratio. Scale bar = 500 μm.

Imaging studies of subcellular distribution

Experimental procedure

Imaging was performed using a Zeiss LSM 710 laser confocal microscope and a 20× objective. The microscope was operated with ZEN 2011 software. An argon ion laser (excitation 458, 488 and 514 nm) was used to visualize doxorubicin and doxorubamine. A diode laser (excitation 405 nm) was used to visualize Hoechst-33342. Regions of interest (ROI) were identified using phase contrast microscopy. To measure the accumulation and localization of doxorubicin or doxorubamine, fluorescence images were acquired using the Alexa Fluor 568 detector. To locate the Hoechst-33342-stained nuclear region, fluorescence images were acquired using the DAPI standard blue channel detector.

In brief, MES-SA cells (MilliporeSigma, catalog # 95051030) were cultured in McCoy's 5A (Modified) Medium supplemented with 10% heat-deactivated fetal bovine serum (FBS) and penicillin/streptomycin (P/S). The cultures were incubated in a humidified atmosphere with 5% CO_2 at 37 °C. Approximately 24 h before imaging, MES-SA cells were plated with 2 mL of medium in glass-bottom imaging dishes coated with poly-D-lysine (MatTek; 35 mm dishes, No. 1.5 coverslip, 14 mm glass diameter). Approximately 12 h before imaging, the dishes were treated with doxorubicin (Dox) or $DoxNH_2NH_2$ (2 µL of 10 mM stock in water; final concentration 10 µM), respectively. One hour prior to imaging, the cells were washed with PBS and the medium in the dish was replaced with Fluorobrite^{TM} Dulbecco's Modified Eagle Medium (DMEM), and the cells were stained with Hoechst 33342 (1 µL; 1 mg/mL stock in water) before being returned to the incubator. Control samples that were not treated with Dox or $DoxNH_2NH_2$ were prepared analogously.

To qualify the subcellular fluorescence distribution, ImageJ 1.54p was used to calculate the background fluorescence of each image by averaging the fluorescence intensity of five approximately cell-sized regions, in which cells or debris were absent. This value was then used to correct the fluorescence intensity measured for the cytoplasm and nucleus in the corresponding image. For all experiments, the quantification was accomplished based on a minimum of 100 cells.

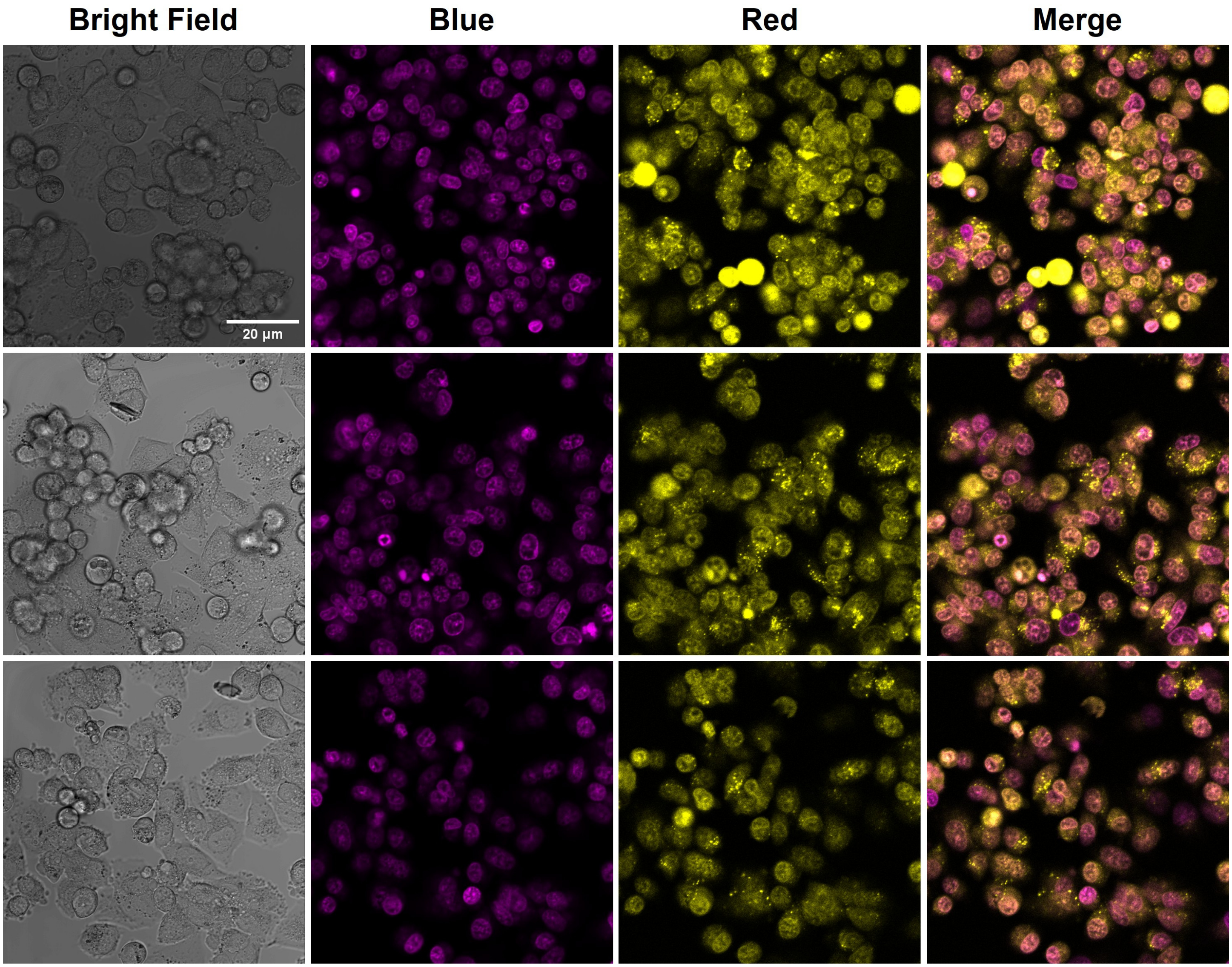

Figure S12. Images from fluorescence microscopy studies showing compound localization in MES-SA cells treated with doxorubicin (10 μM) for 12 h. Image sets are three representative regions from the first plate.

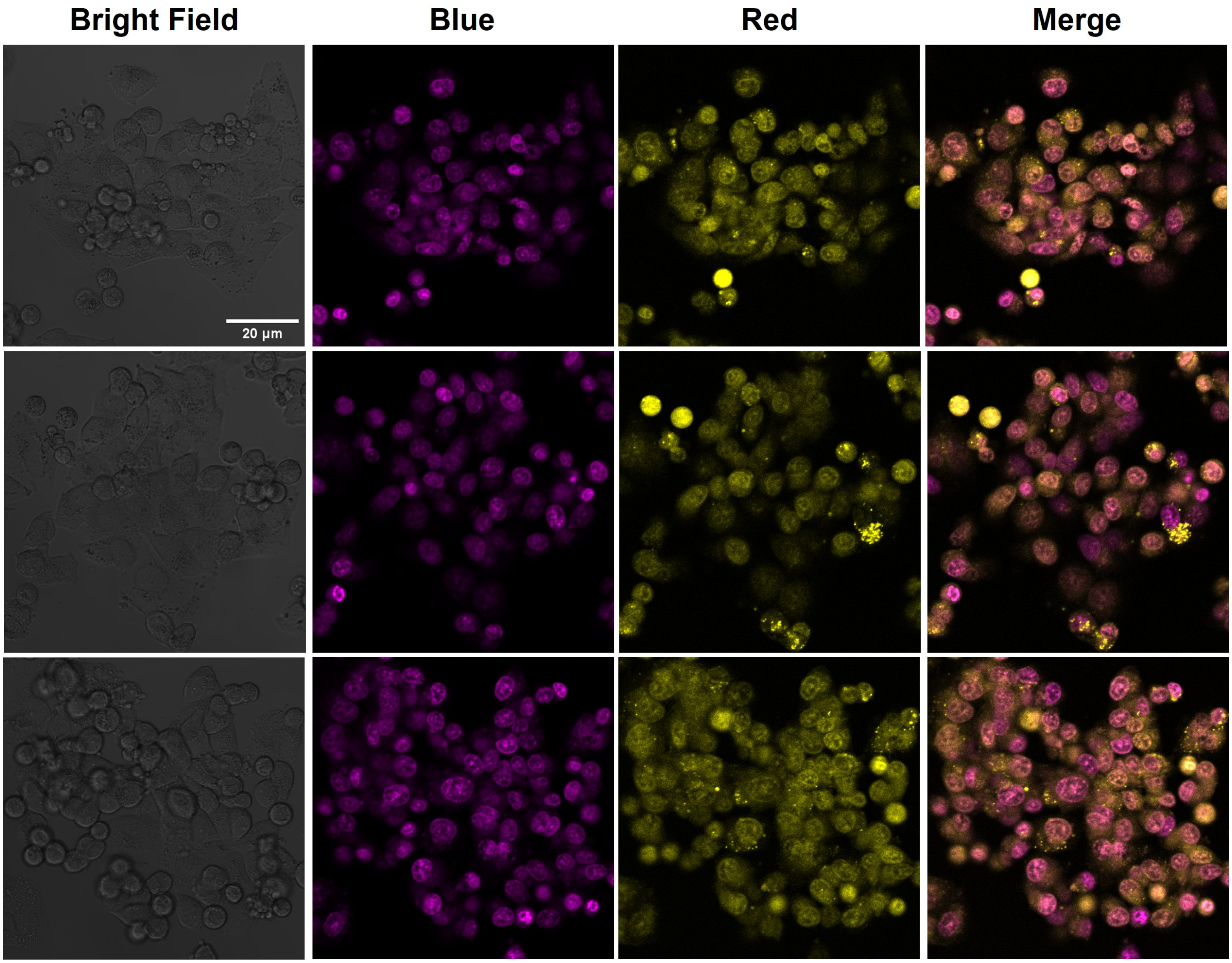

Figure S13. Images from fluorescence microscopy studies showing compound localization in MES-SA cells treated with doxorubicin (10 μM) for 12 h. Image sets are three representative regions from the second plate.

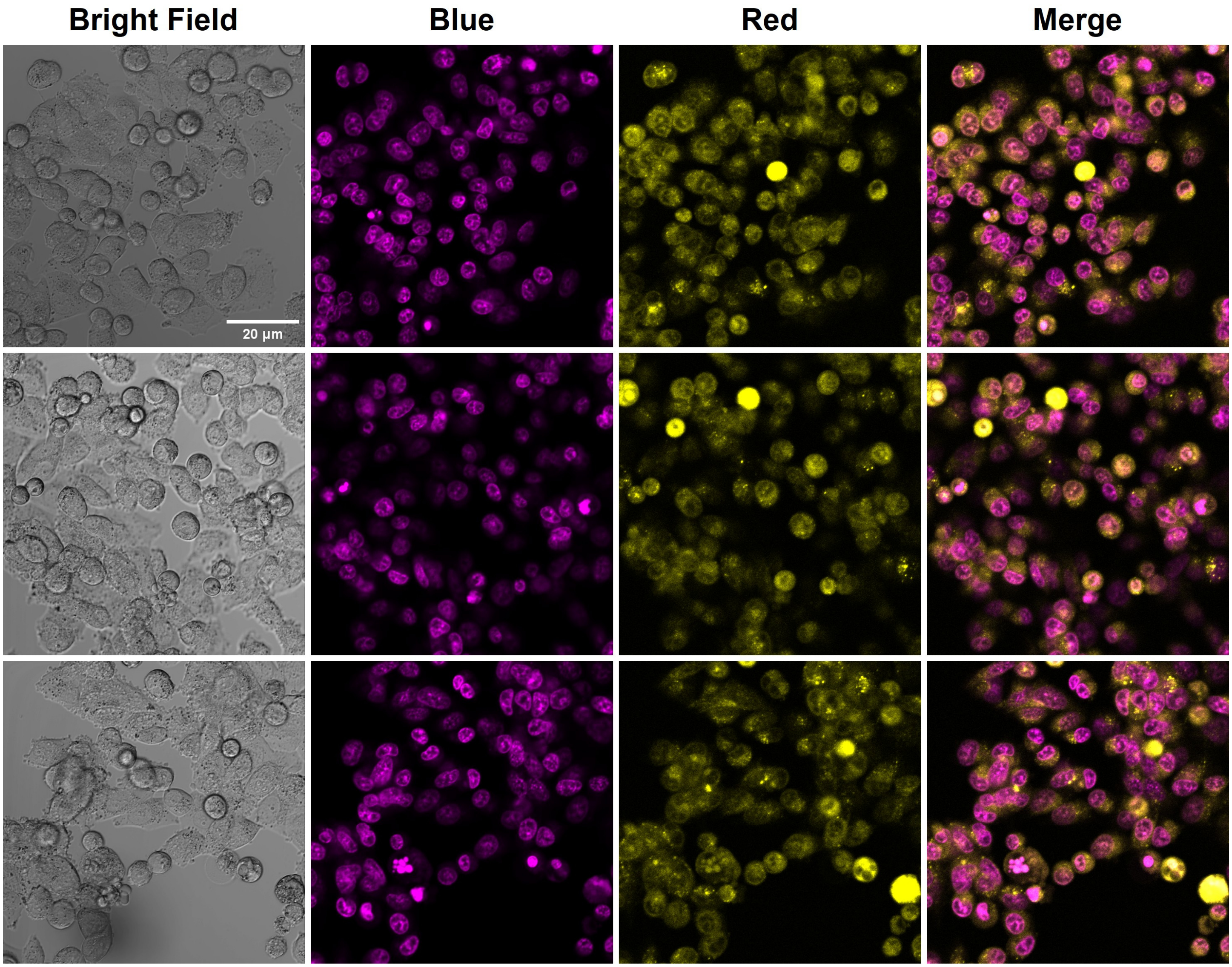

Figure S14. Images from fluorescence microscopy studies showing compound localization in MES-SA cells treated with doxorubamine (10 μM) for 12 h. Image sets are three representative regions from the first plate.

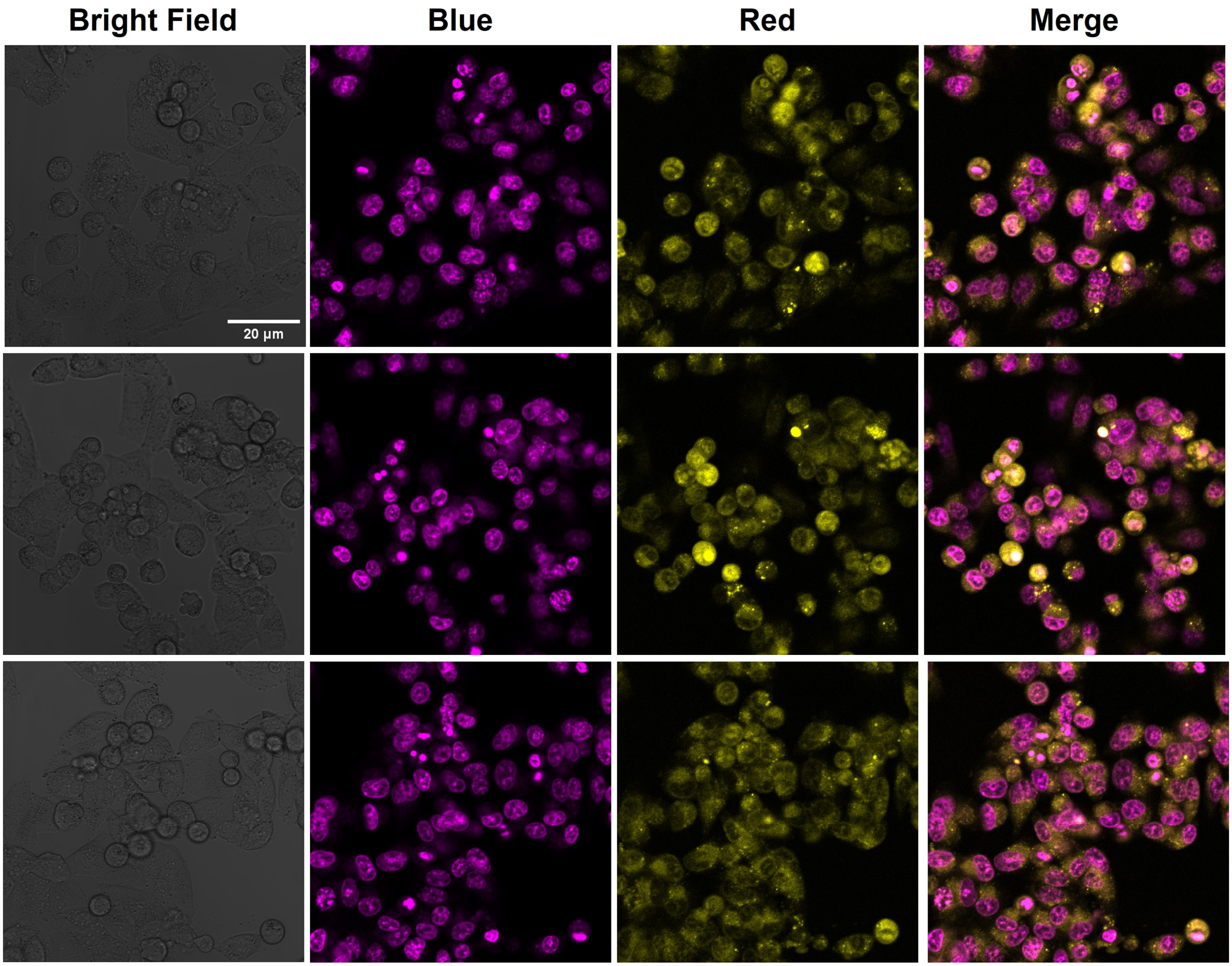

Figure S15. Images from fluorescence microscopy studies showing compound localization in MES-SA cells treated with doxorubamine (10 µM) for 12 h. Image sets are three representative regions from the second plate.

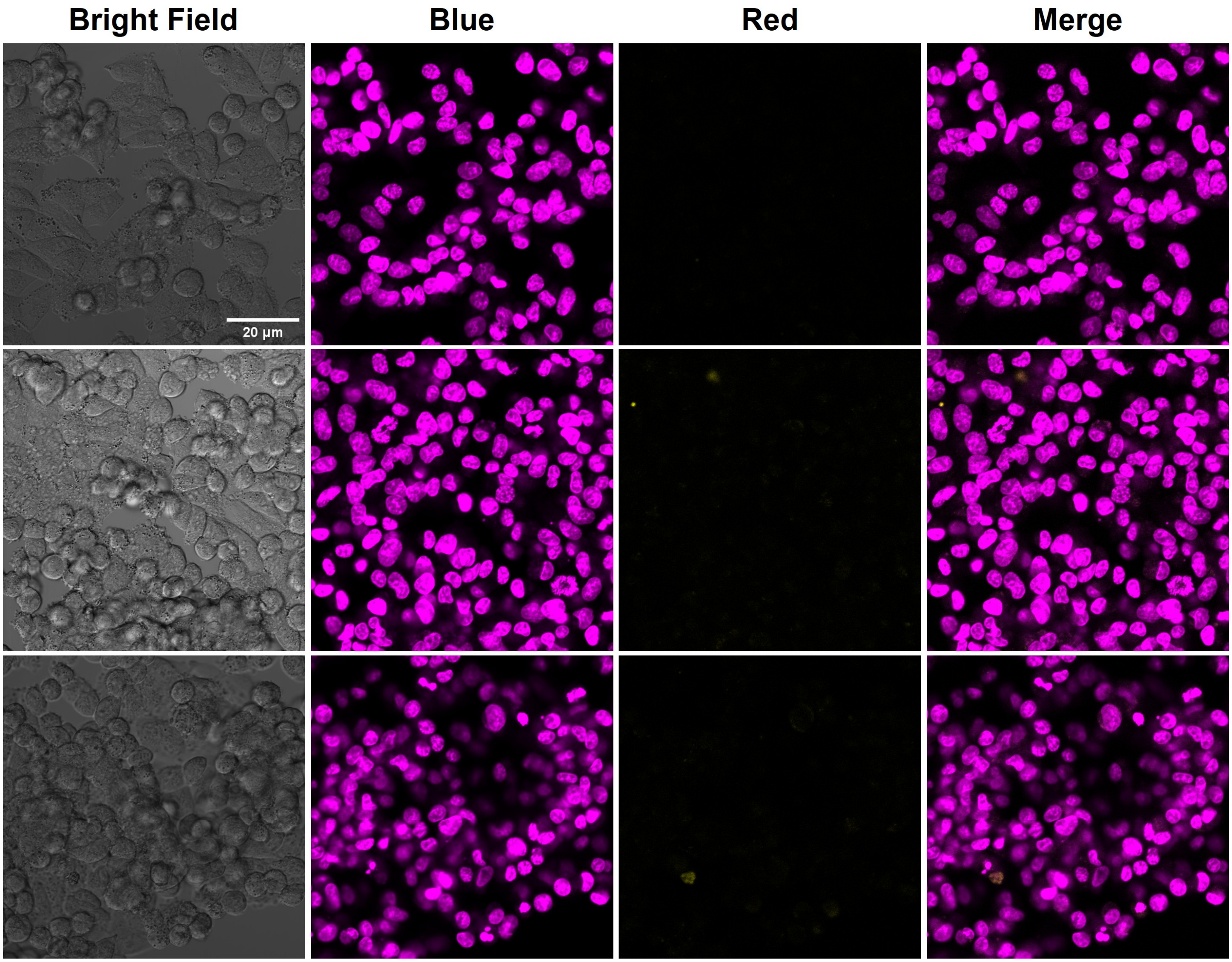

Figure S16. Images from fluorescence microscopy studies of untreated MES-SA cells. Image sets are three representative regions from the first plate.

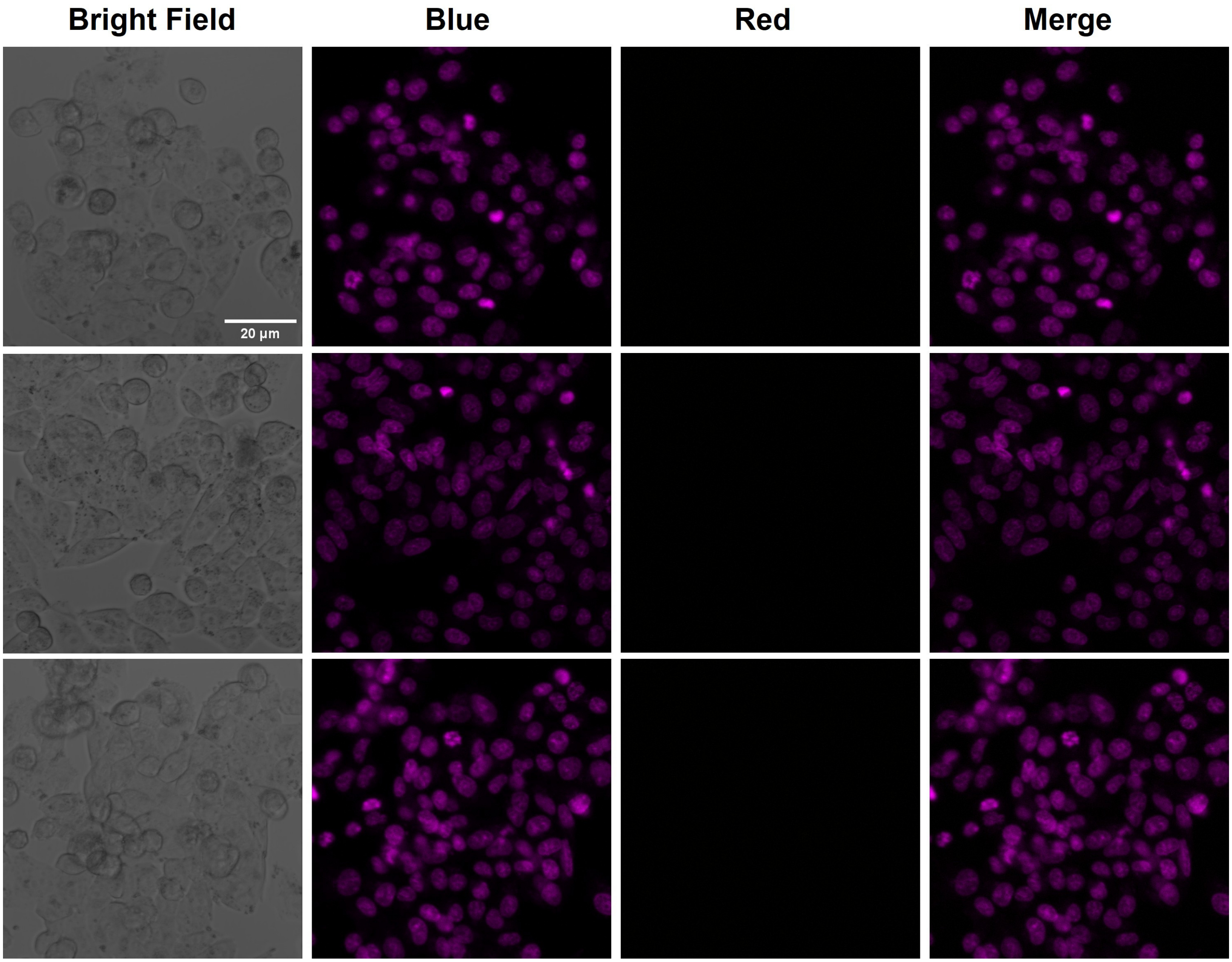

Figure S17. Images from fluorescence microscopy studies of untreated MES-SA cells. Image sets are three representative regions from the second plate.

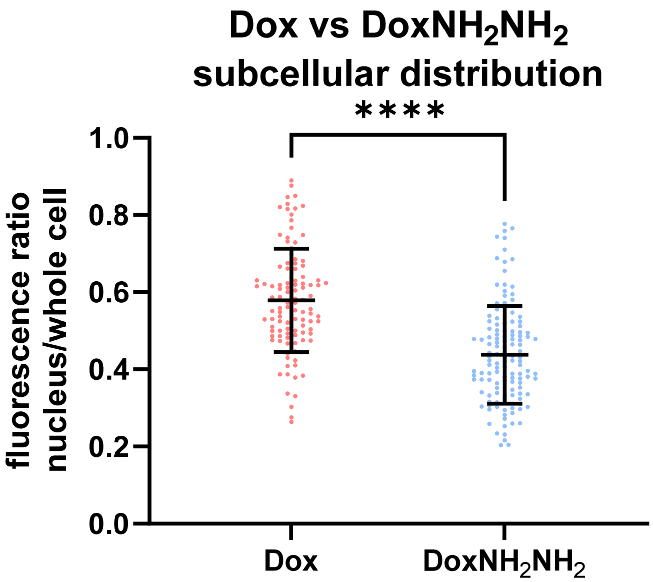

Figure S18. Imaging quantification as measured by the ratio of fluorescence in the nucleus and in the whole cell. ****$P <$ 0.0001 by unpaired two-tailed Welch's t test. For doxorubicin, n = 106 cells. For doxorubamine, n = 128 cells. Error bars represent standard deviations.

Determination of partition coefficient in n-octanol–pH 7.4 phosphate buffer mixture

Water-saturated *n*-octanol was prepared by stirring *n*-octanol with water for 12 h. An aliquot of doxorubicin or doxorubamine stock solution in water (10 µL, 5.0 mM) was added to a mixture of 1× phosphate-buffered saline (PBS, pH 7.4, 190 µL) and water-saturated *n*-octanol (200 µL) in a 0.5 mL Seal-Rite® 0.5 mL microcentrifuge tube. The mixture was shaken on an IKA® MS 3 digital orbital shaker at 3000 rpm for 180 min in the dark. The mixture was transferred to a 1.5 mL microcentrifuge tube and spun at 10000 rpm for 5 min at 24 °C. Aliquots of the aqueous and the *n*-octanol phases (150 µL each) were transferred, respectively, by a micropipette to cuvettes containing methanol and 1× pH 7.4 PBS (2:1 v/v, 1850 µL). The partition coefficient was calculated as the ratio of absorbance determined at 490 nm. This experiment was repeated three times for each compound.

Compound	*P* (Sample 1)	*P* (Sample 2)	*P* (Sample 3)	Average Log *P* and standard deviation
Dox	0.35	0.36	0.38	−0.44±0.01
$DoxNH_2NH_2$	1.1	1.5	1.4	0.12±0.05

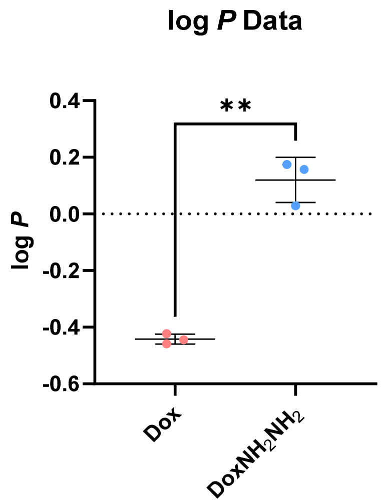

Figure S19. Logarithm of the partition coefficient of Dox and $DoxNH_2NH_2$ in *n*-octanol–pH 7.4 phosphate buffer mixture. Error bars represent standard deviations. ***P* = 0.0048 by unpaired two-tailed Welch's *t* test.

Reference

1. Wang, F.; Braverman, J.; Eng, G.; Leylek, Ö.; Petrone, N. L.; Honeycutt, D. S.; Imada, S.; Pallares, B.; Zhang, D.; Mrosla, J. M., et al., Leveraging platinum-protein interactions to overcome chemoresistance. *Nat. Commun.* **2025,** *16*, 9263.
